# Author Correction: Joint disease-specificity at the regulatory base-pair level

**DOI:** 10.1038/s41467-022-28073-6

**Published:** 2022-01-27

**Authors:** Pushpanathan Muthuirulan, Dewei Zhao, Mariel Young, Daniel Richard, Zun Liu, Alireza Emami, Gabriela Portilla, Shayan Hosseinzadeh, Jiaxue Cao, David Maridas, Mary Sedlak, Danilo Menghini, Liangliang Cheng, Lu Li, Xinjia Ding, Yan Ding, Vicki Rosen, Ata M. Kiapour, Terence D. Capellini

**Affiliations:** 1 Department of Human Evolutionary Biology, Harvard University, Cambridge, MA USA; 2 Department of Orthopedics, Affiliated Zhongshan Hospital of Dalian University, Dalian, China; 3 Department of Orthopaedic Surgery, Boston Children’s Hospital, Harvard Medical School, Boston, MA USA; 4 Department of Developmental Biology, Harvard School of Dental Medicine, Boston, MA USA; 5 Department of Surgery, the Second Affiliated Hospital of Dalian Medical University, Dalian, China; 6 Department of Pediatrics, Boston Children’s Hospital, Harvard Medical School, Boston, MA USA; 7 Broad Institute of MIT and Harvard, Cambridge, MA USA; 8 Present Address: Farm Animal Genetic Resources Exploration and Innovation Key Laboratory of Sichuan Province, Sichuan Agricultural University, Chengdu, China

**Keywords:** Genetics, Musculoskeletal system

Correction to: *Nature Communications* 10.1038/s41467-021-24345-9, published online 6 July 2021.

In this article the funding from ‘the Faculty Council at Boston Children’s Hospital’ was omitted. The original article has been corrected.

