# Supplementary material for: Joint disease-specificity at the regulatory base-pair level
Source: Nat Commun. 2021 Jul 6;12:4161. doi: 10.1038/s41467-021-24345-9 (PMC8260791; doi:10.1038/s41467-021-24345-9)
Supplement: Supplementary file 1 — Supplementary Information [file 41467_2021_24345_MOESM1_ESM.pdf]

Supplementary Information

Supplementary Methods

GWAS analyses on joint disease

In order to retrieve GWAS datasets across multiple musculoskeletal diseases, as well as developmental phenotypes, the MESH disease-term nomenclature was utilized. The MESH ID D009140, 'Disorder of musculoskeletal system', along with D012863, 'Skeleton', were used as top hierarchical terms with which to search for related disease terms. This was done using the 'MeSH.db' library version 1.13.0[1] in R version 3.6.1 (R Development Core Team, 2008) – all daughter terms listed for this ID were obtained, with their corresponding daughter terms iteratively retrieved until the lowest terms of each hierarchy were reached. Subsequently, we manually removed those retrieved MESH IDs representing diseases indirectly linked to bone biology, for example, those originating from a more immunological basis (such as rheumatoid arthritis). While this approach is by no means entirely comprehensive, it allowed for a standardized, relatively unbiased approach at identifying relevant disease terms. This curated set of MESH IDs was then used to query the GWAS Central database[2], retrieving summary-level statistics for significant GWAS lead variants for all studies matching any of these IDs. Similarly, these MESH IDs were mapped to equivalent EFO terms using a Python-based script (https://github.com/warrenread/efo-mesh-mapping) and subsequently used to identify sets of GWAS studies listed in the GWAS Catalog[3], using this database's API in order to extract significant lead variants from these studies. Collected variant information was consolidated between these two databases, with duplicated studies removed. For each study, lead variants were aggregated – in the event that a variant was reported twice in the same study (e.g. for discovery/replication cohorts) only unique variants were retained. Variants were subsequently

pooled across studies, for a final set of 740 significant lead variants. These variants were counted within 50kb windows (defined across the hg38 genome using R) with the bedtools version 2.27.1 'intersect' function[4]. Window counts were plotted along chromosome ideograms using karyoploteR version 1.12.4[5]. Within each chromosome, the set of windows was used to fit a smoothening spline with the 'smooth.spline' function in base R with the degrees-of-freedom set to the number of unique per-window counts and the 'all.knots' option enabled. Fitted values were subsequently overlayed with per-window counts with karyoploteR. These values were also used to define a red-blue density color-scale to indicate the distribution of aggregated GWAS signal along the length of a chromosome; this heatmap was similarly overlayed using karyoploteR.

We next examined the chromosomal regions containing the densest GWAS association signals. A region on chromosome 2 containing the *TGFA-IT1* gene, which has been associated with osteoarthritis risk[6], had GWAS association signals across numerous studies. Another densely replicated region on chromosome 6 contains the genes *TAP1* and *PSMB9*, as do windows on chromosome 15 (*SMAD3*) and chromosome 3 (*PBRM1*, *GNL3*). Another such region, and focus of this study, appears on chromosome 20 at the *GDF5-UQCC1* locus, which is also the area of this chromosome capturing the greatest overlap of GWAS signal. The presence of signals in the *UQCC1* locus fall intronic in previously reported regulatory landscape for *GDF5*[7,8] (Supplementary Data 1). The *GDF5* locus associates with a number of musculoskeletal diseases and traits (Supplementary Table 1). Please also see main text for details.

Supplementary Table 1. Musculoskeletal traits and diseases associated with *GDF5* haplotype

Disease/Trait	Reference*
Height	[30]
Developmental dysplasia of the hip	[22]
Femoral neck size	[31]
Hip bone size	[31]
Hip circumference	[32]
Intertrochanteric region size	[31]
Joint mobility	[33]
Knee pain	[34]
Hip osteoarthritis	[35]
Knee osteoarthritis	[36]
Spine bone size	[31]
Trochanter size	[31]
Waist-hip ratio	[37]
Hip axis length	[38]
**Lumbar disc degeneration	[39]
**Lower back pain	[40]
** Lumbar disc herniation	[40]
** Achilles tendinopathy	[41]
** Meniscus injury	[42]
** Temporomandibular joint osteoarthritis	[43]
** Hand osteoarthritis	[44]
** Non-contact anterior cruciate ligament rupture	[45]

* - Example references provided

** - Candidate association studies or metanalyses

ATAC-seq analyses on E67 acetabulum, femur and tibia samples:

Developmental dysplasia of the hip (DDH) is of developmental origin, and chondrogenesis during hip formation has a major role in sculpting femoral head and neck and acetabular morphology[9]. While knee OA occurs late in life, it is known to be the result of altered chondrocyte biology, and to this end we recently reported enrichments of GWAS variants in knee developmental chondrocyte open chromatin regions[10]. Finally, we and others have reported on the importance of *GDF5* to the development of both joints[7,8,10-20]. Collectively, these findings support examining developmental tissues to find regulatory regions in the *GDF5* locus and beyond involved in each respect joint's formation and disease risk. To this end, we first used the assay for transposase accessible chromatin followed by sequencing (ATAC-seq) on *in vivo* E67 human embryonic acetabulum, proximal and distal femoral and proximal and distal tibial chondrocytes (see Methods) to identify accessible or "open" chromatin sequences. We then acquired stage-matched mouse E15.5 data on a subset of the same structures (proximal and distal femur and proximal and distal tibia) from Richard et al[10].

After procuring human samples (see Methods) (n = 3/tissue/timepoint), and performing ATAC-seq protocol on each of five tissues (i.e., acetabulum, proximal femur, distal femur, proximal tibia and distal tibia), we used the Irreproducible Discovery Rate (IDR) method[21] to call reproducible peaks or putative regulatory regions across the three replicates per tissue. IDR peak calls per tissue are provided in Supplementary Data 2. We then identified human and mouse ATAC-seq peaks within the knee OA and DDH association interval in the *GDF5-UQCC1* locus, albeit some flanking gene transcription start site (TSS) regions show accessible chromatin signals (Fig. 1, Supplementary Fig. 2). These accessibility signals at the locus overlap with previously reported E59 ATAC-seq data from the femur and tibia[10]. We also used this

accessibility data to explore patterns of modularity and multiple-disease risk associations on a genome-wide level, as discussed later.

ATAC-seq peak and risk variant intersection to whittle-down the *GDF5-UQCC1* DDH and knee OA association intervals:

To identify putatively causal variant(s) for DDH and knee OA in the *GDF5* locus, we obtained GWAS variants from three large studies, two DDH GWAS, one on Europeans[22] and the other in Asians[23] (Supplementary Data 3), and a recent knee OA GWAS conducted in Europeans[6]. For the European DDH GWAS, 23 variants were in strong linkage disequilibrium (LD >0.90) with the lead rs143384 variant. For the Asian DDH GWAS, 11 variants were in strong linkage disequilibrium (i.e., LD >0.98) to a different downstream lead variant (rs6060373) (Supplementary Data 3). Comparison of both DDH GWAS, 7 DDH variants were shared between both population sets (Supplementary Data 3), reflecting a shorter risk haplotype in Asians spanning downstream regulatory regions in the locus. The lead knee OA variant (rs143384) was in linkage disequilibrium with 95 fine-mapped variants, present in the 95% CI credible set[6] (Supplementary Data 3).

To help reduce these variant lists to fewer putative causal DDH and knee OA variants, we intersected all three GWAS variant sets with human embryonic E67 ATAC-seq peaks from the tissues described above, and orthologous mouse E15.5 ATAC-seq peaks from similar tissues curated from Richard *et al* [10] (Supplementary Data 3). We intersected them with the locations of previously identified *GDF5* enhancers (i.e., *GROW1*[7] and *R1-R5*[8]). As reported[7,8], these enhancers were originally identified via a tiling screen across the >100 kb *GDF5-UQCC1* locus screening human and mouse sequence in fosmid and plasmid lacZ reporter-based assays *in vivo*.

We also used an additional published ATAC-seq dataset on patient osteoarthritic proximal tibia from knees[24] albeit this was based on a limited replicate size. We also intersected these variants with GTEx eQTL datasets. GTEx data used for the analyses described in this manuscript were obtained from the GTEx Portal on 03/17/21. The Genotype-Tissue Expression (GTEx) Project was supported by the Common Fund of the Office of the Director of the National Institutes of Health, and by NCI, NHGRI, NHLBI, NIDA, NIMH, and NINDS.

DDH variant intersections

Beginning with the European DDH GWAS variant set, we identified 2 variants intersecting human acetabulum and/or proximal femur ATAC-seq regions (rs4911178, rs6060369). We also identified the same 2 European variants (rs6060369, rs4911178) intersecting proximal femur ATAC-seq regions in orthologous mouse sequence (Supplementary Data 3). For the Asian DDH GWAS variant set, we identified 3 variants intersecting human acetabulum and/or proximal femur ATAC-seq regions (rs4911178, rs6060355, rs6088791). We also identified 1 Asian variant (rs4911178) intersecting ATAC-seq regions in orthologous mouse proximal femur sequence (Supplementary Data 3). Therefore, of the 7 shared European/Asian DDH GWAS variants (Supplementary Data 3), only 1 shared variant (rs4911178) resides in acetabulum and/or proximal femur ATAC-seq regions in humans, and this is also the same variant residing in relevant mouse ATAC-seq regions.

The multiple overlapping signals that rs4911178 has with regulatory regions involved in both human and mouse hip development permits its functional testing i*n vitro* (in human cells) as well as *in vivo* (in the mouse model). As DDH is a three-dimensional disease that emerges during

embryonic/fetal life, the functional orthology of the *GROW1* enhancer in both humans and mice makes rs4911178 a priority candidate to study. However, here we note the following details about the two additional intersecting Asian DDH variants (rs6060355 and rs6088791) detected above: (1) Regarding rs6060355, this variant resides in a previously described *GDF5* regulatory element reported as the *R5* enhancer[7,8]. Using transgenic reporter assays, we found that the *R5* enhancer, as well as the broader human 35 kb fosmid sequence containing *R5*, drove *lacZ* expression in and below the knee and not in the proximal femur or acetabulum of the hip. Therefore, the ATAC-seq region harboring this variant likely does not reflect an active enhancer in the hip. We also examined GTEx eQTL data and found that this variant acts as an eQTL for *UQCC1* (exposed skin), *EDEM2* (transformed fibroblasts), and *GDF5* (pituitary). (2) Regarding rs6088791, this variant is immediately adjacent to the *R4* enhancer (and rs6060369, see below), which as we show in the main text has no effects on the hip-joint when excised from the mouse. It was determined as a variant intersecting the accessibility over the *R4* element, and not as a separate signal. Our analysis of GTEx data reveals that this variant acts as an eQTL for *UQCC1* (lung, esophageal mucosa, cultured fibroblasts), *ERGIC3* (brain hippocampus), and *GDF5* (lung).

Knee OA variant intersections

Continuing with the knee OA GWAS variant set, we intersected it with human skeletal E67 ATAC-seq data, and identified 7 variants intersecting human distal femur and/or proximal tibia ATAC-seq regions (rs6060369, rs6088791, rs4911491, rs735531, rs4911178, rs6088816, rs143383). We then identified only two variants (rs6060369, rs4911178) intersecting ATAC-seq regions in orthologous mouse ATAC-seq sequence. Comparing both sets of intersections, we found only two variants that consistently fell within functionally conserved human and mouse

skeletal ATAC-seq regions (rs6060369, rs4911178) (Supplementary Data 3). For the knee OA patient ATAC-seq dataset, we identified 6 variants (rs6060369, rs6088791, rs6060373, rs4911491, rs735531, rs143383)(Supplementary Data 3), of which 5 variants (rs6060369, rs6088791, rs4911491, rs735531, and rs143383) consistently intersected ATAC-seq signals from both developmental and aging ATAC-seq datasets, and of these, only 1 (rs6060369) overlaps ATAC-seq regions in mouse. Similar to the DDH intersections above, we also performed an analysis of knee OA variants overlapping the locations of previously identified human/mouse *GDF5* skeletal enhancers. Of the knee OA variants, only two resided within known enhancers (rs4911178 in the *GROW1* and rs6060369 in the *R4*) or near an enhancer (rs6088791 near the *R4* enhancer).

Due to the multiple overlapping signals that rs6060369 has with regulatory regions involved in skeletal development and aging biology/disease, that these are shared between humans and mouse, and that we recently functionally characterized this variant in knee OA *in vitro* and i*n vivo*[10], we focus on this variant in the main text, and its role in adult mouse knee biology, patient knee OA risk, and hip biology. However, here we report on the other variants (rs6088791, rs4911491, rs735531, rs143383) which intersect with or are directly adjacent to ATAC-seq regions/*GDF5* enhancers: (1) Regarding rs6088791, the variant is immediately adjacent to the *R4* enhancer and is not contained within the functional enhancer element proper. Our analysis of GTEx data reveals that this variant acts as an eQTL for *UQCC1* (lung, esophageal mucosa, cultured fibroblasts), *ERGIC3* (brain hippocampus), and *GDF5* (lung). (2) Regarding rs4911491, this variant resides in previously analyzed sequence (termed Region 4) experimentally tested in Capellini et al[7] *in vivo,* which was shown to drive no reproducible expression in the skeleton. Therefore, the element is likely not an enhancer. The variant also is noted as acting as an GTEx

eQTL only for the intronic gene *UQCC1* across a seven non-skeletal tissues (subcutaneous adipose, visceral adipose (omentum), tibial nerve, tibial artery, pancreas, skin (non-exposed), stomach (REF). (3) Regarding rs735531, this variant also resides in Region 4, and acts as an GTEx eQTL for *UQCC1* (whole blood, lung, esophageal mucosa, and cultured fibroblasts), *ERGIC3* (brain hippocampus), and *GDF5* (lung). (4) Regarding rs143383, this variant resides in the *GDF5* 5'UTR, and overlaps accessibility data because of its proximity to *GDF5/Gdf5* promoter, which is consistently marked due to active gene transcription. This variant acts as an eQTL for *UQCC1* (whole blood) and *GDF5* (lung).

DDH patient rs4911178 allele and genotype analyses:

Patient DNA samples (n = 113; see Methods) were PCR amplified to detect the rs4911178 variant. Sanger sequencing was then used to detect which allele(s) (i.e., non-risk G, or risk A) were present at rs4911178, and each patient's allele (G or A) and genotype (G/G, G/A, A/A) was tallied (Supplementary Data 4). TDC and YD reviewed all chromatograms and determined allelic composition for each patient sample (blinded to ID and prior to any morphological analyses). Allele frequency and genotype frequency in the patient cohort was then calculated. Given the practical difficulty of having patient matched controls for childhood DDH, we made use of the 1000 Genomes Project data as controls, notably the allele and genotype frequencies in three Chinese populations: Han Chinese in Beijing, China (CHB), Han Chinese South (CHS), and the Chinese Dai in Xishuangbanna, China (CDX), with the CHB being most proximate to our patient sample. Allele and genotype frequencies were calculated for each of three general population control samples (1000 Genomes Project, CHB (n = 103), CHS (n = 105), and CDX (n =93), with the CHB showing the highest "A" allele and "A/A" genotype frequencies. We then used the Chi-Square statistic to compare observed to expected allele and genotype frequencies

between the DDH and CHB samples, with 1 degree of freedom for two alleles, or 2 degrees of freedom for three genotypes. Given differences in sample size between DDH (113) and CHB (103) we performed statistical analyses in two ways, each showing similar results. First, we compared data from DDH and 1000 Genomes Project individuals directly not accounting for differences in samples size (e.g., 113 DDH patient set versus a 103 CHB sample set). Second, we compared data from DDH and 1000 Genomes Project individuals by recalculating allele and genotype counts using the observed allele frequency of the "A" variant and genotype frequency of the "A/A" genotype in the CHB set but scaled to 113 individuals (to match the sample number of DDH patients). Results of these analyses are presented in Supplementary Data 4.

DDH patient morphometric analyses:

In order to confirm our preclinical observations of vertically inclined acetabula, as the hallmark of DDH, in *GROW1* and rs4911178 mice, we quantified acetabular inclination angle of Sharp from X-Ray images in the DDH cohort. We first compared several measures (acetabulum inclination angle of sharp, acetabular index (Tönnis angle) and center-edge angle acetabular inclination angle), between patients who are "A/A" risk allele homozygous and those who are "A/G" risk heterozygous, noting that there were only 4 "G/G" non-risk individuals in this set. We then compared acetabulum inclination angle of sharp measured from the DDH cohort with published normative values on subjects from China (n = 204), Japan (n = 254), and Korea (n = 591). The results showed significantly more vertical acetabula (Sharp angle of 46.5 +/- 3.2) in DDH patients compared to Chinese (37.8 +/- 4.4; $P<0.001$), Japanese (37.9 +/- 3.8; $P<0.001$) and Korean (37.1 +/- 4; $P<0.001$) normal controls. This is in complete agreement with preclinical observations of more vertical acetabulum in *GROW1* (wild type: 76.9 +/- 4.8; homozygous: 83.5 +/- 6.0; $P=0.021$) and humanized rs4911178 (wild type: 67.2 +/- 3.7; homozygous: 74.6 +/- 1.8;

P<0.001) mice. The combination of these clinical and preclinical observations highlights the important role of *GROW1* enhancer and T risk allele in the pathomechanism of DDH.

Supplementary Table 2. Number of analyzed mice for each line.

Line	Timepoint	Wild type	Heterozygous	Homozygous
$GROW1^{+/-}$	P30	7	12	12
$GROW1^{rs4911178\text{-}A/rs4911178\text{-}+}$	P56	12	44	14
$R4^{+/-}$	P30	5	5	5
$R4^{+/-}$	P365	6	15	14
$R4^{rs6060369\text{-}T/rs6060369\text{-}+}$	P56	6	8	6
$R4^{rs6060369\text{-}T/rs6060369\text{-}+}$ (Bony Morphometry)	P365	12	19	22
$R4^{rs6060369\text{-}T/rs6060369\text{-}+}$ (OARSI Scoring)	P365	5		10

$GROW1^{-/-}$ and rs4911178 single base-pair replacement mice morphometric analyses:

At P30, mice with homozygous removal of the *GROW1* enhancer had significantly shallower trochlear groove, shorter femoral neck, smaller femoral head diameter, and smaller, more vertical and more anteverted acetabula, compared to the wild types (Fig. 2c and Supplementary Tables 3, 4, 5). These changes correspond to the location of *GROW1* expression in pelvis, proximal femur and distal femur growth plate (Fig. 2b). There were no differences in valgus cut angle, neck-shaft

angle, femoral head offset, femoral neck diameter, and acetabular depth as well as quantified features of the femoral condyles, tibial plateau (Supplementary Fig. 3 and Supplementary Tables 3, 4 and 5).

Supplementary Table 3. *p*-values for pairwise comparisons in pelvis anatomy in *GROW1* enhancer knockout mice at P30. ANOVA with Dunnet post-hoc was used for pairwise comparisons to wild type (control). All *p*-values are two-sided.

Anatomical Feature	HET vs WT	HOMO vs WT
Acetabular Diameter	0.084	0.002
Acetabular Depth	0.999	0.953
Acetabular Inclination	0.546	0.021
Acetabular Version	0.815	0.004

WT: Wild type; HET: Heterozygous; HOMO: Homozygous

Supplementary Table 4. *p*-values for pairwise comparisons in tibia anatomy in *GROW1* enhancer knockout mice at P30. ANOVA with Dunnet post-hoc was used for pairwise comparisons to wild type (control). All *p*-values are two-sided.

Anatomical Feature	HET vs WT	HOMO vs WT
Tibial Width	0.996	0.371
Medial tibial spine height	0.986	0.142
Lateral tibial spine height	0.988	0.437
Medial tibial slope	0.344	0.823
Lateral tibial slope	0.282	0.998

WT: Wild type; HET: Heterozygous; HOMO: Homozygous

Supplementary Table 5. *p*-values for pairwise comparisons in femur anatomy in *GROW1* enhancer knockout mice at P30. ANOVA with Dunnet post-hoc was used for pairwise comparisons to wild type (control). All *p*-values are two-sided.

Anatomical Feature	HET vs WT	HOMO vs WT
Femoral Bicondylar width	0.898	0.181
Femoral Intercondylar notch width	0.561	0.368
Medial femoral condyle width	0.985	0.778
Lateral femoral condyle width	0.999	0.739
Medial femoral condyle curvature radius	0.999	0.918
Lateral femoral condyle curvature radius	0.459	0.893
Medial Trochlear Width	0.771	0.262
Central Trochlear Width	0.853	0.470
Lateral Trochlear Width	0.367	0.022
Trochlear Depth	0.883	0.027
Trochlear Sulcus Angle	0.911	0.003
Valgus Cut Angle	0.401	0.358
Neck Shaft Angle	0.355	0.916

Femoral Neck Length	0.069	0.004
Femoral Neck Diameter	0.739	0.829
Femoral Head Offset	0.835	0.555
Femoral Head Diameter	0.027	0.003

WT: Wild type; HET: Heterozygous; HOMO: Homozygous

At P56, mice with homozygous risk allele ("A/A") at rs4911178 had significantly larger valgus cut angle, larger neck-shaft angle, and smaller, more vertical and more anteverted acetabula, compared to the wild types (Fig. 2d and Supplementary Tables 6, 7 and 8). These changes correspond to the location of *GROW1* expression in pelvis and proximal femur (Fig. 2b). There were no differences in femoral head offset and diameter, acetabular depth, as well as quantified features of distal femur and proximal tibia (Supplementary Fig. 4 and Supplementary Tables 6, 7 and 8).

Supplementary Table 6. *p*-values for pairwise comparisons in pelvis anatomy in rs4911178 variant mice at P56. ANOVA with Dunnet post-hoc was used for pairwise comparisons to wild type (control). All *p*-values are two-sided.

Anatomical Feature	HET vs WT	HOMO vs WT
Acetabular Diameter	0.979	0.002
Acetabular Depth	0.462	0.903
Acetabular Inclination	0.397	<0.001
Acetabular Version	0.076	<0.001

WT: Wild type; HET: Heterozygous; HOMO: Homozygous

Supplementary Table 7. *p*-values for pairwise comparisons in tibia anatomy in rs4911178 variant mice at P56. ANOVA with Dunnet post-hoc was used for pairwise comparisons to wild type (control). All *p*-values are two-sided.

Anatomical Feature	HET vs WT	HOMO vs WT
Tibial Width	0.210	0.183
Medial tibial spine height	0.469	0.797
Lateral tibial spine height	0.997	0.593
Medial tibial slope	0.613	0.960
Lateral tibial slope	0.954	0.967

WT: Wild type; HET: Heterozygous; HOMO: Homozygous

Supplementary Table 8. *p*-values for pairwise comparisons in femur anatomy in rs4911178 variant mice at P56. ANOVA with Dunnet post-hoc was used for pairwise comparisons to wild type (control). All *p*-values are two-sided.

Anatomical Feature	HET vs WT	HOMO vs WT
Femoral Bicondylar width	0.929	0.926
Femoral Intercondylar notch width	0.984	0.923
Medial femoral condyle width	0.108	0.355
Lateral femoral condyle width	0.113	0.143
Medial femoral condyle curvature radius	0.840	0.328
Lateral femoral condyle curvature radius	0.742	0.952
Medial Trochlear Width	0.823	0.817
Central Trochlear Width	0.914	0.972
Lateral Trochlear Width	0.104	0.733
Trochlear Depth	0.053	0.636
Trochlear Sulcus Angle	0.414	0.979
Valgus Cut Angle	0.040	0.013
Neck Shaft Angle	0.001	<0.001

Femoral Neck Length	0.945	0.881
Femoral Neck Diameter	0.107	0.989
Femoral Head Offset	0.865	0.911
Femoral Head Diameter	0.927	0.971

WT: Wild type; HET: Heterozygous; HOMO: Homozygous

PITX1 ChIP assay:

In silico analyses revealed that compared to the non-risk "G" variant, the "A" variant at rs4911178 reduces predicted binding by the PITX1 transcription factor. PITX1 is a master regulator of hind limb development, expressed in hind limb joints and growth plates, and whose coding and non-coding mutations in humans, mice, and other vertebrates, such as stickleback fish, lead to significant pelvic and knee defects, along with shortened hindlimbs[25-29]. To go beyond computational predictions and directly demonstrate that PITX1 is bound to *GROW1*, we performed chromatin immunoprecipitation (ChIP) experiments in both human and mouse chondrocytes. First, similar to *R4*[10], we found that PITX1 is bound to *GROW1* in human T/C-28a2 chondrocytes (Fig.3d). We next assessed whether PITX1 was bound *in vivo* in mouse chondrocytes extracted from the developing wildtype proximal femur during a gestational period when the *GROW1* element was active (e.g., E15.5). Indeed, we found significant binding of PITX1 to the *GROW1* enhancer *in vivo* (Fig.3e), with the strongest binding observed for the proximal femur, followed by the distal femur, and then proximal tibia. In order to determine whether PITX1 is differentially bound at the orthologous rs4911178 G/A position, we performed ChIP on *GROW1* $^{rs4911178\text{-}A/rs4911178\text{-}G}$ mice and then used MiSeq on pulled-down amplicons to determine if there is an allelic imbalance due to different binding affinities. When normalized by

input ratios, the DDH risk "A" variant decreased binding of PITX1 by 39.6 % in the proximal femur (n=3, p= 0.0022), 34.5 % in the distal femur (n=3, p=0.0023), and 20.2 % in the proximal tibia (n=3, p = 0.0015), relative to PITX1 binding at the wildtype "G" variant in each tissue (Fig.3f). These findings are in line with strongest changes in *GDF5* expression observed in the proximal femur, compared to other joint sites. Overall, this decreased binding of PITX1 to the "A" variant at the orthologous rs4911178 base-position, coupled with decreased *GDF5* expression demonstrates a molecular mechanism of action of the variant change at the locus.

Rs6060369 allele in knee OAI patients:

OAI individuals were grouped based on the severity of OA presenting in either knee at baseline (KL grade) into two groups: KL0-1 and KL>=2. Genotyping data for these individuals was used to count the occurrence of the 'T' risk allele at rs6060369 in either group using a down-sampling strategy (see Methods). Sampling (n = 200) those individuals entering the OAI study already exhibiting OA phenotypes (KL >=2) in at least one knee we observed a significantly greater frequency of the 'T' risk allele (254.5 +/- 0.66) compared to sampled individuals presenting with KL0-1 (245.8 +/- 0.70) (p-value: 1.17e-11).

OAI patient rs6060369 morphometric analyses:

Compared to wild types ("C/C"), OAI individuals with homozygous risk allele ("T/T") at the rs6060369 locus had smaller knees (bicondylar width and tibial plateau width), smaller femoral condyles (medial and lateral) and curvier medial femoral condyles (Fig. 4b; Supplementary Table 9). These are the same trends seen in mice with homozygous risk alleles "T/T" at rs6060369 locus (Fig. 4c; Supplementary Tables 13 and 14). There was a trend towards more

vertical ACLs in homozygous patients, compared to wild types, which approached statistical significance ($p = 0.074$; Supplementary Table 9).

Supplementary Table 9. *p*-values for pairwise comparisons in knee anatomy in OAI subjects. ANOVA with Dunnet post-hoc was used for pairwise comparisons to wild type (control). All *p*-values are two-sided.

Anatomical Feature	HET vs WT	HOMO vs WT
Femoral Bicondylar width	0.754	0.009
Femoral Intercondylar notch width	0.312	0.810
Medial femoral condyle width	0.694	0.029
Lateral femoral condyle width	0.606	0.005
Medial femoral condyle curvature radius	0.895	0.038
Lateral femoral condyle curvature radius	0.611	0.213
Tibial plateau width	0.393	0.002
Medial tibial spine height	0.243	0.968
Lateral tibial spine height	0.369	0.405
Coronal tibial slope	0.316	0.765
Medial tibial slope	0.341	0.174

Lateral tibial slope	0.563	0.977
Medial tibial depth	0.295	0.588
ACL length	0.608	0.248
ACL sagittal angle	0.757	0.074
Medial meniscus posterior cross-sectional area	0.982	0.713
Lateral meniscus posterior cross-sectional area	0.565	0.251
Medial meniscus posterior angle	0.736	0.255
Lateral meniscus posterior angle	0.540	0.210

WT: Wild type; HET: Heterozygous; HOMO: Homozygous

***R4**$^{-/-}$* **and rs6060369 single base-pair replacement mice morphometric analyses:**

Despite previously reported significant differences in knee morphology[10], at P30 and P365, there were no differences in proximal femur and pelvis morphology between mice with homozygous removal of the *R4* enhancer compared to wild type controls (Supplementary Tables 10 and 11). Similar trends were also observed in mice with homozygous risk allele at rs6060369 at P56 (Supplementary Table 12). This is consistent with locations where *R4* enhancer is not active[10].

Supplementary Table 10. *p*-values for pairwise comparisons in proximal femur and pelvis anatomy in *R4* enhancer knockout mice at P30. ANOVA with Dunnet post-hoc was used for pairwise comparisons to wild type (control). All *p*-values are two-sided.

Anatomical Feature	HET vs WT	HOMO vs WT
Valgus Cut Angle	0.666	0.620
Neck Shaft Angle	0.965	0.436
Femoral Neck Diameter	0.918	0.434
Femoral Head Offset	0.716	0.356
Femoral Head Diameter	0.272	0.756
Acetabular Diameter	0.808	0.108
Acetabular Depth	0.871	0.987
Acetabular Inclination	0.783	0.612
Acetabular Version	0.893	0.893

WT: Wild type; HET: Heterozygous; HOMO: Homozygous

Supplementary Table 11. *p*-values for pairwise comparisons in proximal femur and pelvis anatomy in *R4* enhancer knockout mice at 1 year. ANOVA with Dunnet post-hoc was used for pairwise comparisons to wild type (control). All *p*-values are two-sided.

Anatomical Feature	HET vs WT	HOMO vs WT
Valgus Cut Angle	0.839	0.999
Neck Shaft Angle	0.969	0.973
Femoral Neck Diameter	0.986	0.104
Femoral Head Offset	0.582	0.322
Femoral Head Diameter	0.930	0.265
Acetabular Diameter	0.477	0.614
Acetabular Depth	0.393	0.878
Acetabular Inclination	0.680	0.569
Acetabular Version	0.725	0.868

WT: Wild type; HET: Heterozygous; HOMO: Homozygous

Supplementary Table 12. *p*-values for pairwise comparisons in proximal femur and pelvis anatomy in rs6060369 variant mice at P56. ANOVA with Dunnet post-hoc was used for pairwise comparisons to wild type (control). All *p*-values are two-sided.

**Anatomical Feature**	**HET vs WT**	**HOMO vs WT**
Valgus Cut Angle	0.456	0.999
Neck Shaft Angle	0.292	0.594
Femoral Neck Length	0.849	0.464
Femoral Neck Diameter	0.134	0.809
Femoral Head Offset	0.861	0.594
Femoral Head Diameter	0.985	0.629
Acetabular Diameter	0.875	0.999
Acetabular Depth	0.799	0.999
Acetabular Inclination	0.667	0.637
Acetabular Version	0.687	0.681

WT: Wild type; HET: Heterozygous; HOMO: Homozygous

At 1-year, mice with homozygous risk allele (“T/T”) at rs6060369 locus had significantly smaller and curvier femoral condyles, smaller notch, smaller tibial spines and steeper tibial plateaus, compared to wild type controls (Fig. 4c; Supplementary Tables 13 and 14). This is

consistent with anatomical differences previously reported in *R4* null mice (at P30 and 1-year) as well as the locations where *R4* enhancer activity is detected.[9] Similar to observations in *R4* null mice, mice with homozygous risk allele in rs6060369 locus had no differences in pelvis and proximal femur anatomy, compared to wild types at 1-year (Supplementary Tables 14 and 15).

Supplementary Table 13. *p*-values for pairwise comparisons in tibia anatomy in rs6060369 variant mice at 1-year. ANOVA with Dunnet post-hoc was used for pairwise comparisons to wild type (control). All *p*-values are two-sided.

**Anatomical Feature**	**HET vs WT**	**HOMO vs WT**
Tibial Width	0.982	0.137
Medial tibial spine height	0.947	0.003
Lateral tibial spine height	0.992	0.010
Medial tibial slope	0.242	<0.001
Lateral tibial slope	0.948	0.009

WT: Wild type; HET: Heterozygous; HOMO: Homozygous

Supplementary Table 14. *p*-values for pairwise comparisons in femur anatomy in rs6060369 variant mice at 1-year. ANOVA with Dunnet post-hoc was used for pairwise comparisons to wild type (control). All *p*-values are two-sided.

**Anatomical Feature**	**HET vs WT**	**HOMO vs WT**
Femoral Bicondylar width	0.513	0.025
Femoral Intercondylar notch width	0.079	<0.001
Medial femoral condyle width	<0.001	<0.001
Lateral femoral condyle width	<0.001	<0.001
Medial femoral condyle curvature radius	0.709	0.026
Lateral femoral condyle curvature radius	0.154	<0.001
Medial Trochlear Width	0.627	0.994
Central Trochlear Width	0.974	0.850
Lateral Trochlear Width	0.826	0.914
Trochlear Depth	0.789	0.984
Trochlear Sulcus Angle	0.073	0.684
Valgus Cut Angle	0.796	0.876
Neck Shaft Angle	0.679	0.947

Femoral Neck Length	0.788	0.735
Femoral Neck Diameter	0.848	0.671
Femoral Head Offset	0.997	0.954
Femoral Head Diameter	0.968	0.939

WT: Wild type; HET: Heterozygous; HOMO: Homozygous

Supplementary Table 15. *p*-values for pairwise comparisons in pelvis anatomy in rs6060369 variant mice at 1-year. ANOVA with Dunnet post-hoc was used for pairwise comparisons to wild type (control). All *p*-values are two-sided.

**Anatomical Feature**	**HET vs WT**	**HOMO vs WT**
Acetabular Diameter	0.991	0.973
Acetabular Depth	0.939	0.983
Acetabular Inclination	0.997	0.749
Acetabular Version	0.994	0.841

WT: Wild type; HET: Heterozygous; HOMO: Homozygous

rs6060369 single base-pair replacement mice histological analyses at 1 year:

Please see main text, Methods, and Supplementary Table 2 (above).

Gene expression and anatomical shape correlations:

Using our mice lines harboring the heterozygous and homozygous genetic variants in the joint enhancers (*GROW1* and *R4*) as well as single base-pair changes resembling the GWAS variants in rs4911178 and rs6060369 variants, we investigated the associations between reductions in *Gdf5* expression and average change in relevant anatomical features. To do so, we use linear regression to test the associations between percent reduction in *Gdf5* expression levels, measured by ASE, and average percent change in relevant anatomical features (i.e. hip morphology for *GROW1* and rs4911178 mice and knee morphology for *R4* and rs6060369 mice) as measured from micro CT images. Results showed a strong association (Fig. 5) between reduction in GDF5 expression and average change in anatomy, which further highlight the altered joint shape as a potential mechanism for GDF5 related joint disease. The regression coefficient (β) was used to find the relative effect size of the reduction on *Gdf5* expression on changes in anatomy. The ratio of average changes in anatomy in homozygous vs heterozygous mutations was calculated to find the relative influence of homozygosity on joint morphology. We observed a strong linear relationship between the level of *Gdf5* expression and extent of change in clinically relevant anatomical features, with higher reduction in *Gdf5* expression leading to larger morphological changes (Fig. 5a). On average, for every 1% reduction in *Gdf5* expression, there was a 0.57% change in anatomy (Figure 5a). Moreover, homozygosity at *R4* or *GROW1* variants resulted in 1.7 (+/- 0.5) fold greater changes in anatomy compared to heterozygous mutations (Figure 5b).

Supplementary Figures

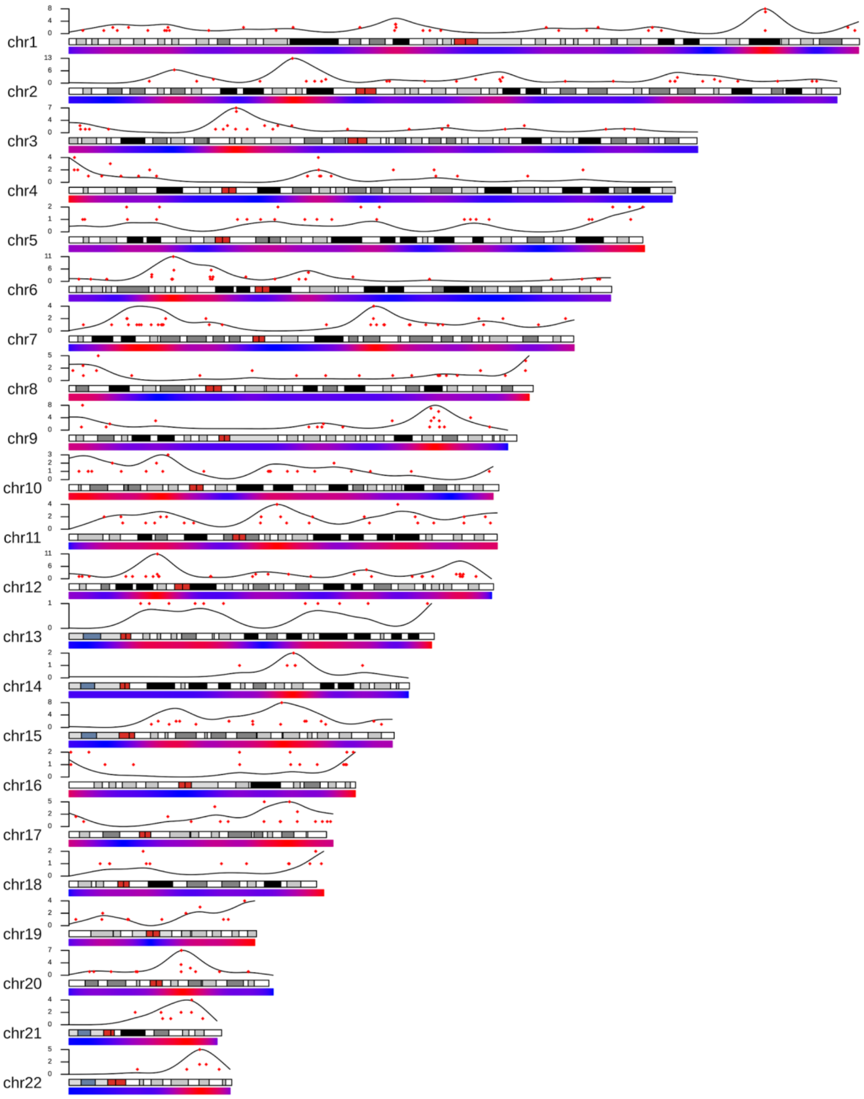

Supplementary Fig. 1 GWAS signals for musculoskeletal disorders. Significant lead variants for an aggregation of musculoskeletal disorders were counted in 50kb windows along all autosomal chromosomes as indicated in red points (left-axis), with smoothened counts density overlaid for the length of each chromosome (top) and along a red-blue color scale (below, normalized per-chromosome). See Methods.

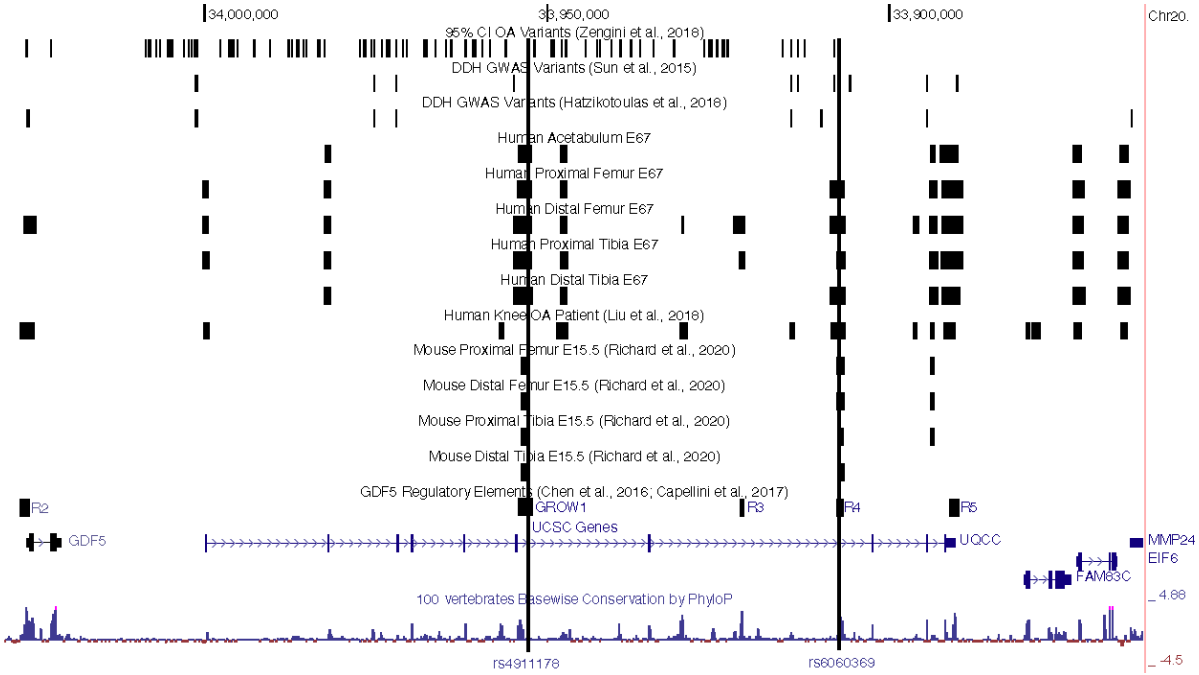

Supplementary Fig. 2 Intersection of human/mouse regulatory sequences with the DDH and knee OA risk variants. Modified hg19 UCSC Genome Browser view showing curated list of European knee OA GWAS variants, Asian DDH GWAS variants, and European DDH GWAS variants (top three rows), human E67 ATAC-seq regions (acetabulum, proximal femur, distal femur, proximal tibia)(next five rows), human knee OA patient ATAC-seq regions (next row), mouse E15.5 ATAC-seq regions (proximal femur, distal femur, proximal tibia, distal tibia)(next four rows), previously published *GDF5* regulatory sequences (next row), UCSC genes (next row), and phyloP100ways vertebrate conservation (final bottom row). Two variants (rs4911178 and rs6060369) overlap with functional regulatory sequences in both human and mouse as highlighted with long vertical black bars.

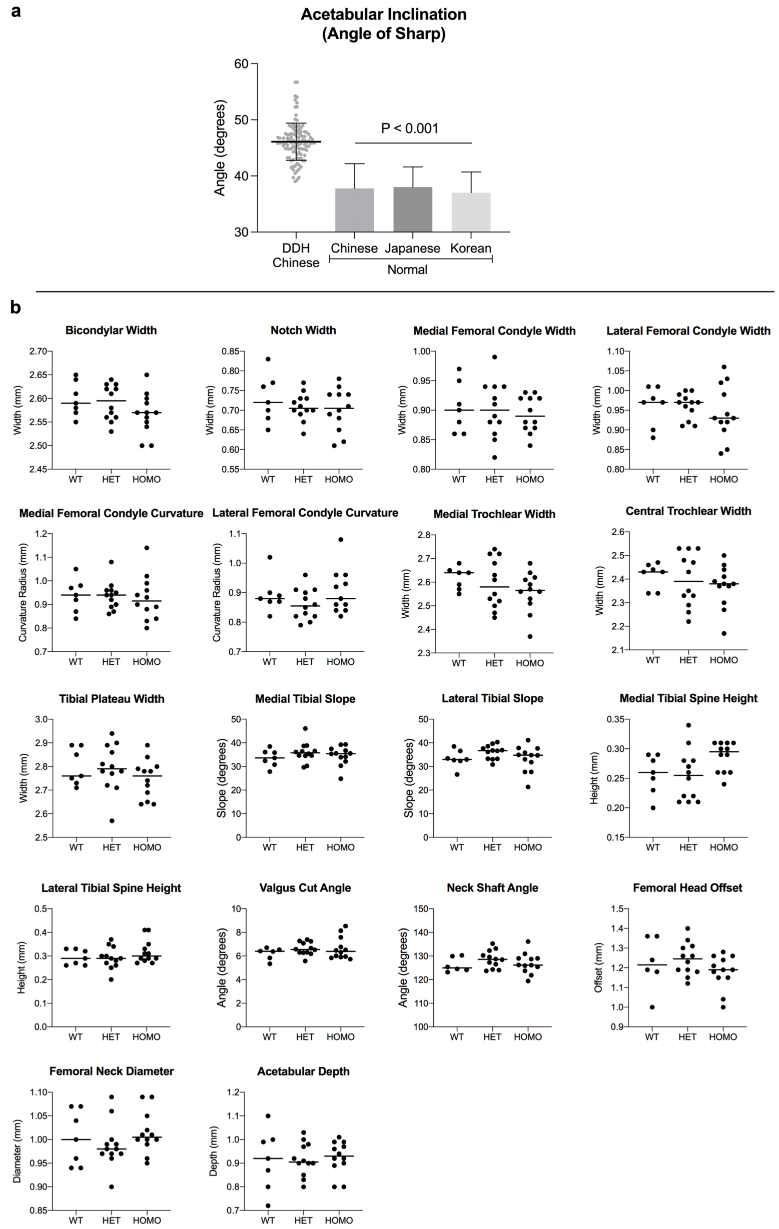

Supplementary Fig. 3 Anatomical features significantly different between DDH patients and population controls; and anatomical features which were not significantly different between genotypes in *GROW1* enhancer null mice. a, Higher acetabular inclination angle in DDH patients compared to controls (DDH Chinese n = 113, Normal Chinese n = 204, Normal Japanese n = 254, Normal Korean n = 591). Independent t-tests were used to compare each normal group to DDH cohort (P<0.0001 for all comparisons) *p*-values are two-sided. The results are shown as mean and standard deviations. The data are also included in the Supplementary Information. b, Number of measurements were carried out in P30 *GROW1* enhancer null mice (see Fig. 2). This figure displays only those which revealed no significant differences between control and *GROW1* enhancer null mice in acetabulum, femur and tibia (WT n = 7, HET n = 12, HOMO n = 12). ANOVA with Dunnet post-hoc was used for pairwise comparisons to wild type. Bars indicate medians.

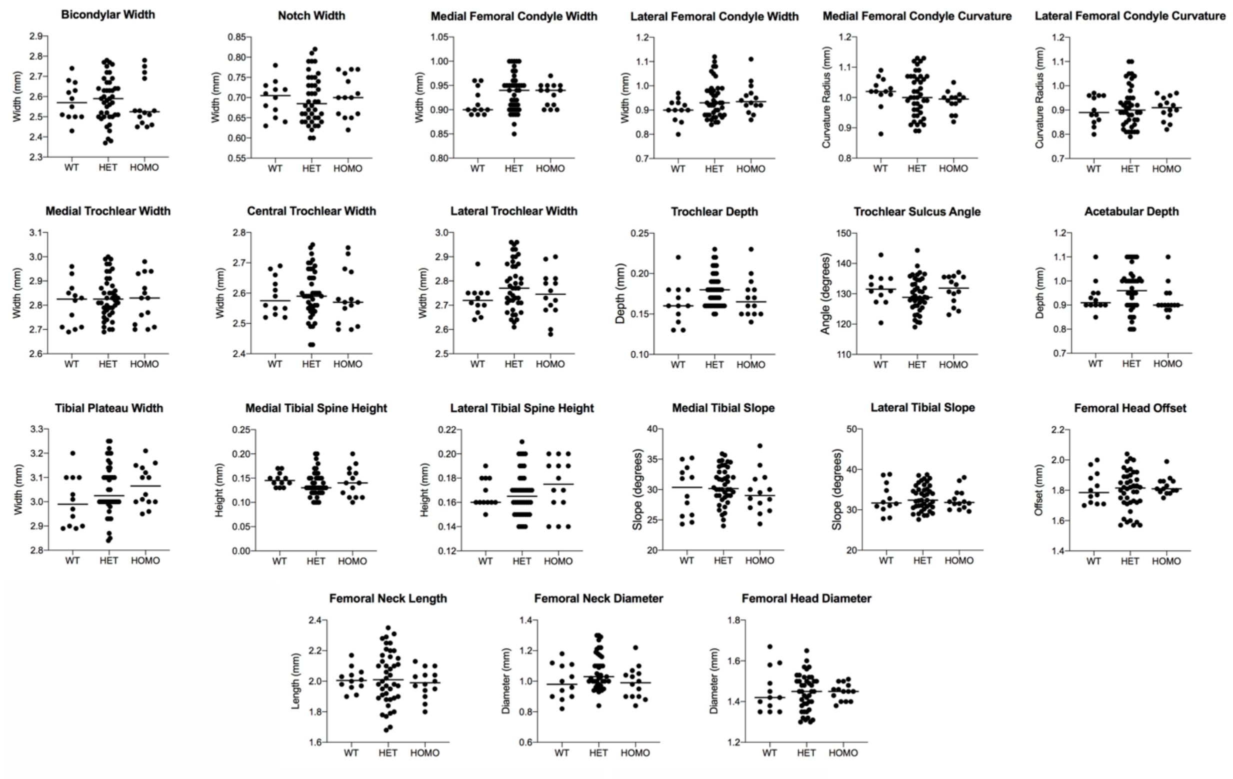

Supplementary Fig. 4 Anatomical features which were not significantly different between genotypes in *GROW1* single allelic replacement mice. A number of measurements were carried out in P56 *GROW1* single allelic replacement mice (see Fig. 2). This figure displays only those which revealed no significant differences between wild type control (*GROW1*$^{rs4911178\text{-}G/rs4911178\text{-}G}$) and *GROW1* single allelic replacement mice (Heterozygous, *GROW1*$^{rs4911178\text{-}A/rs4911178\text{-}G}$ and Homozygous, *GROW1*$^{rs4911178\text{-}A/rs4911178\text{-}A}$) in acetabulum, femur and tibia (WT n = 12, HET n = 42, HOMO n = 14). ANOVA with Dunnet post-hoc was used for pairwise comparisons to wild type. Bars indicate medians.

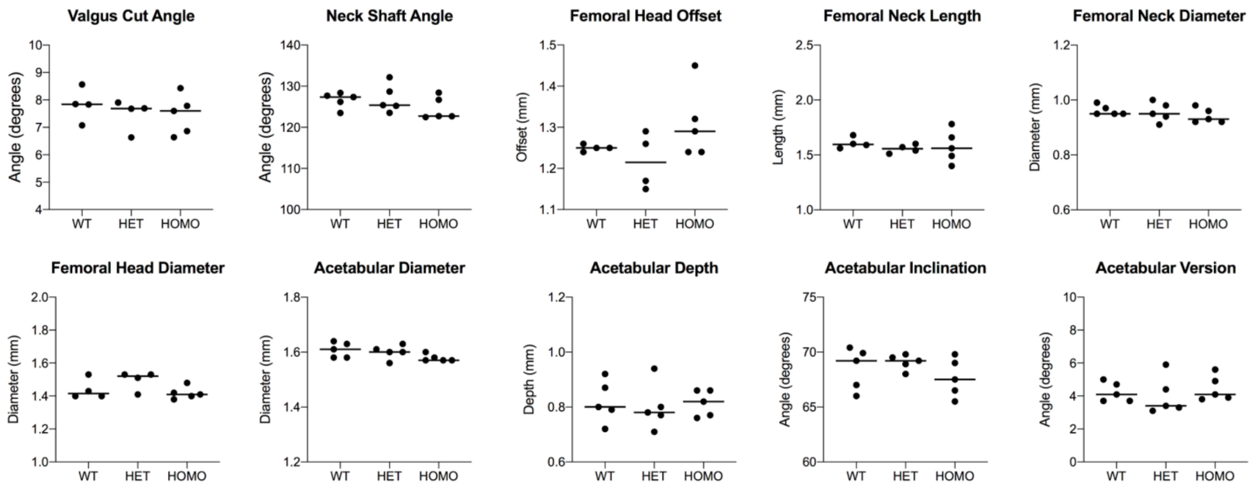

Supplementary Fig. 5 Anatomical features which were not significantly different between genotypes in *R4* enhancer null mice. A number of measurements were carried out in P30 *R4* enhancer null mice. This figure displays only those which revealed no significant differences between control and *R4* enhancer null mice in hip (proximal femur and acetabulum). Bars indicate medians (n = 5 per group). ANOVA with Dunnet post-hoc was used for pairwise comparisons to wild type.

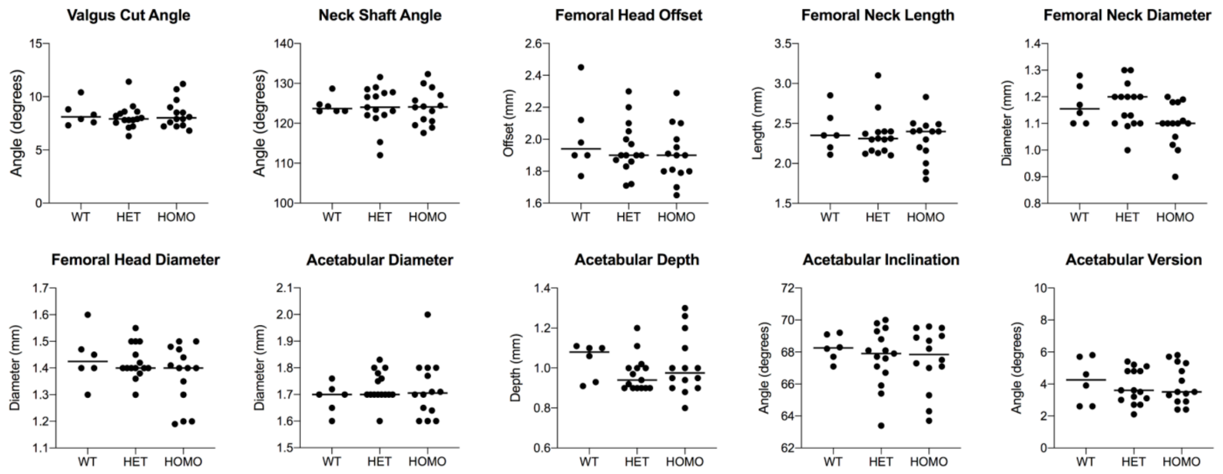

Supplementary Fig. 6 Anatomical features which were not significantly different between genotypes in *R4* enhancer null mice. A number of measurements were carried out in 1-year *R4* enhancer null mice. This figure displays only those which revealed no significant differences between control and *R4* enhancer null mice in hip (proximal femur and acetabulum). Bars indicate medians (WT n = 6, HET n = 14, HOMO n = 14). ANOVA with Dunnet post-hoc was used for pairwise comparisons to wild type.

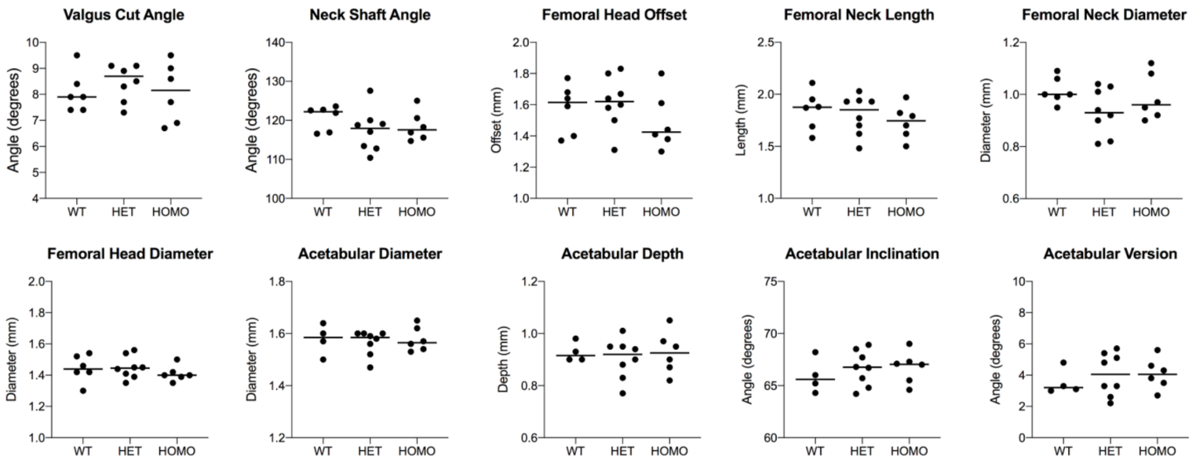

Supplementary Fig. 7 Anatomical features which were not significantly different between genotypes in *R4* single allelic replacement mice. A number of measurements were carried out in P56 *R4* single allelic replacement mice. This figure displays only those which revealed no significant differences between wild type control ($R4^{rs6060369\text{-}A/rs6060369\text{-}A}$) and *R4* single allelic replacement mice (Heterozygous, $R4^{rs6060369\text{-}A/rs6060369\text{-}T}$ and Homozygous, $R4^{rs6060369\text{-}T\,/rs6060369\text{-}T}$) in hip (proximal femur and acetabulum). Bars indicate medians (n = 6 per group). ANOVA with Dunnet post-hoc was used for pairwise comparisons to wild type.

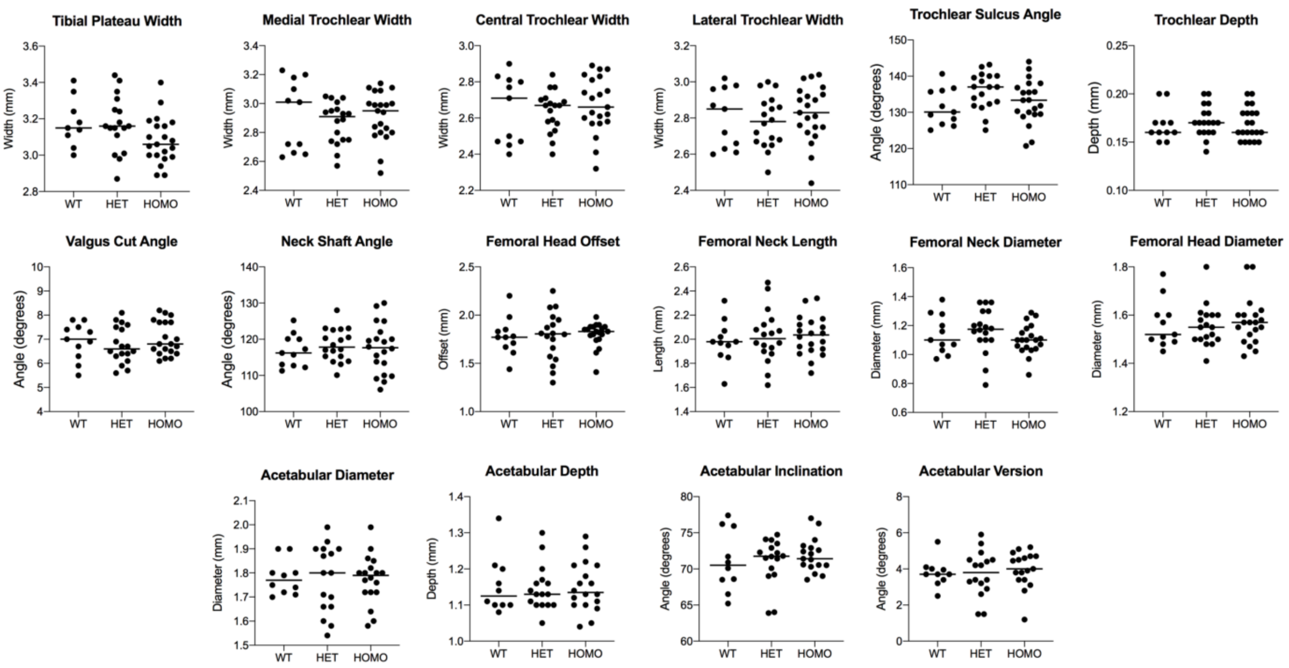

Supplementary Fig. 8 Anatomical features which were not significantly different between genotypes in 1-year *R4* single allelic replacement mice. A number of measurements were carried out in 1-year *R4* single allelic replacement mice (see Fig.5). This figure displays only those which revealed no significant differences between wild type control ($R4^{rs6060369\text{-}A/rs6060369\text{-}A}$) and *R4* single allelic replacement mice (Heterozygous, $R4^{rs6060369\text{-}A/rs6060369\text{-}T}$ and Homozygous, $R4^{rs6060369\text{-}T/rs6060369\text{-}T}$) in femur and tibia. Bars indicate medians (WT n = 11, HET n = 18, HOMO n = 20). ANOVA with Dunnet post-hoc was used for pairwise comparisons to wild type.

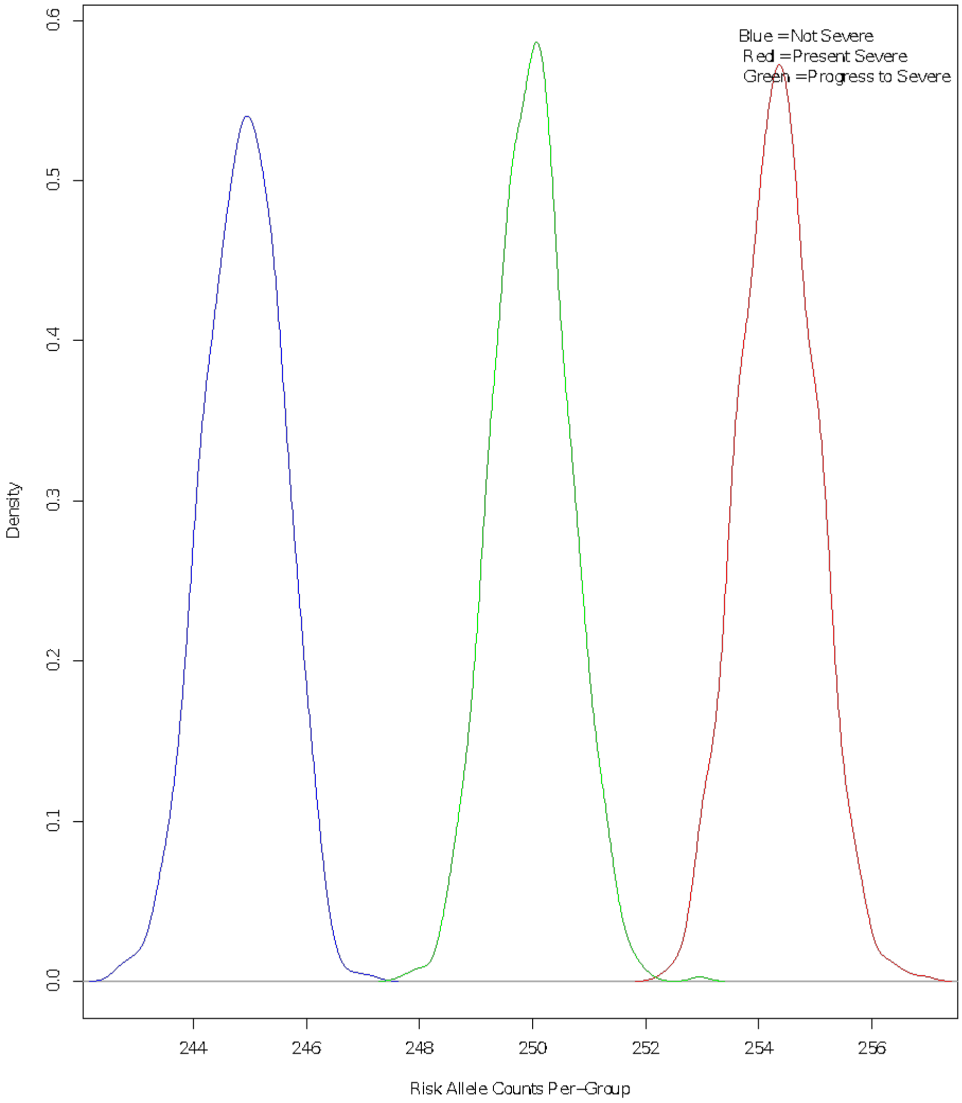

Supplementary Fig. 9 Density plot showing the progression of OA in OAI cohort. Occurrence of the rs6060369 risk “T” allele were counted for subsamples (n = 200) of the OAI cohort, stratified by OA progression. Subsampled density of risk-allele counts are shown for those never presenting with, presenting with, or progressing to severe OA (blue, red and green, respectively). See Supplementary Data 5.

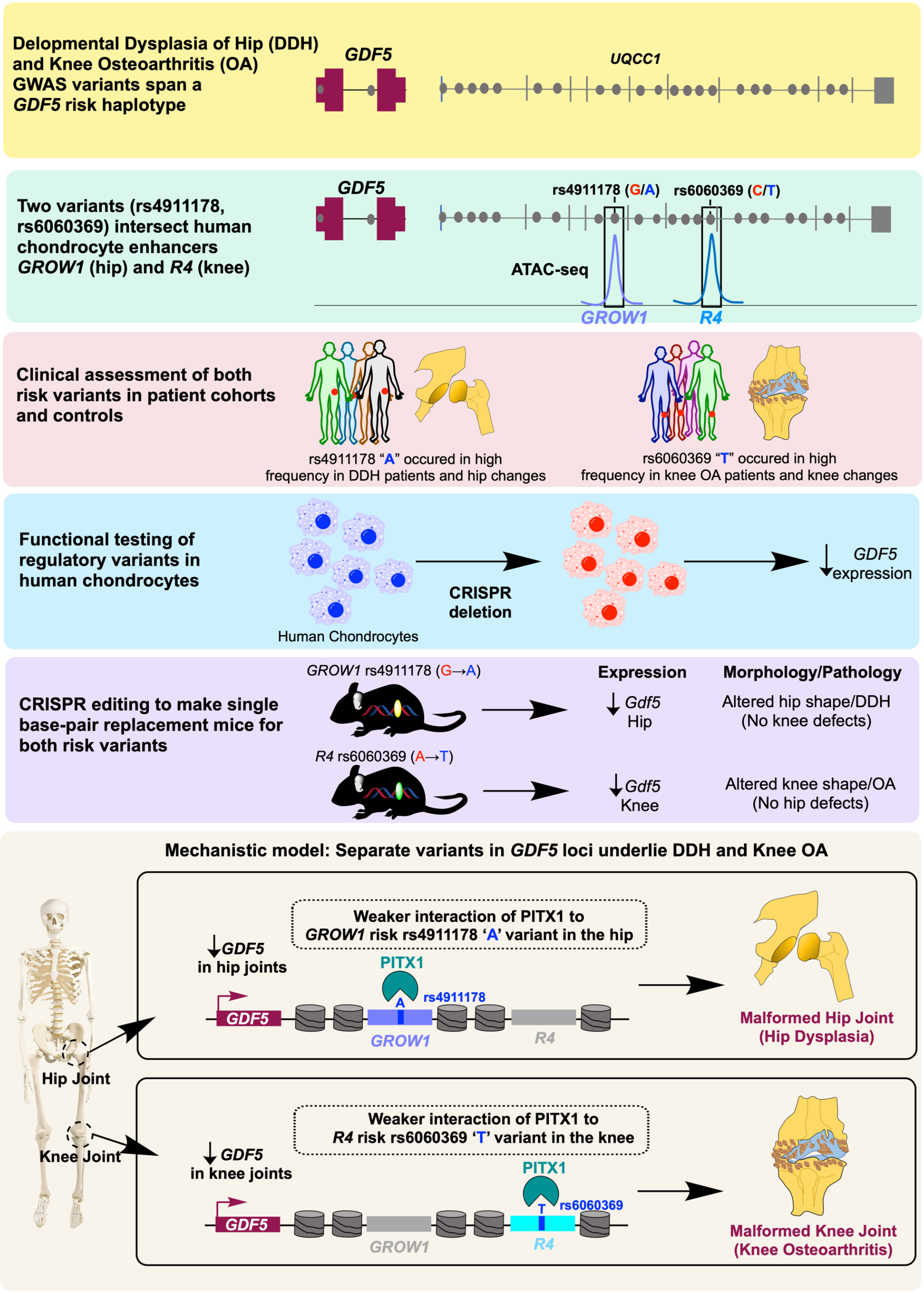

Supplementary Fig. 10 Summary of steps involved in uncoupling regulatory risk variants that underlie DDH and knee OA. Using a combination of functional genomics on human developmental skeletal tissues, assessment of relevant clinical measurements on imaging data from human patients and humanized mice, and targeted genetics in the mouse model, we demonstrate for each disease that genetic variants on the same risk *GDF5* haplotype can be functionally uncoupled and impact bone shape, and disease etiology, in a joint-specific manner.

References

1 Tsuyuzaki, K. *et al.* MeSH ORA framework: R/Bioconductor packages to support MeSH over-representation analysis. *BMC Bioinformatics* **16**, 45, doi:10.1186/s12859-015-0453-z (2015).

2 Beck, T., Shorter, T. & Brookes, A. J. GWAS Central: a comprehensive resource for the discovery and comparison of genotype and phenotype data from genome-wide association studies. *Nucleic acids research* **48**, D933-D940, doi:10.1093/nar/gkz895 (2020).

3 Buniello, A. *et al.* The NHGRI-EBI GWAS Catalog of published genome-wide association studies, targeted arrays and summary statistics 2019. *Nucleic acids research* **47**, D1005-D1012, doi:10.1093/nar/gky1120 (2019).

4 Quinlan, A. R. & Hall, I. M. BEDTools: a flexible suite of utilities for comparing genomic features. *Bioinformatics (Oxford, England)* **26**, 841-842, doi:10.1093/bioinformatics/btq033 (2010).

5 Gel, B. & Serra, E. karyoploteR: an R/Bioconductor package to plot customizable genomes displaying arbitrary data. *Bioinformatics (Oxford, England)* **33**, 3088-3090, doi:10.1093/bioinformatics/btx346 (2017).

6 Zengini, E. *et al.* Genome-wide analyses using UK Biobank data provide insights into the genetic architecture of osteoarthritis. *Nat Genet* **50**, 549-558, doi:10.1038/s41588-018-0079-y (2018).

7 Capellini, T. D. *et al.* Ancient selection for derived alleles at a GDF5 enhancer influencing human growth and osteoarthritis risk. *Nat Genet* **49**, 1202-1210, doi:10.1038/ng.3911 (2017).

8 Chen, H. *et al.* Heads, Shoulders, Elbows, Knees, and Toes: Modular Gdf5 Enhancers Control Different Joints in the Vertebrate Skeleton. *PLoS Genet* **12**, e1006454, doi:10.1371/journal.pgen.1006454 (2016).

9 Wilkinson, J. M. & Zeggini, E. The Genetic Epidemiology of Joint Shape and the Development of Osteoarthritis. *Calcif Tissue Int*, doi:10.1007/s00223-020-00702-6 (2020).

10 Richard, D. *et al.* Evolutionary Selection and Constraint on Human Knee Chondrocyte Regulation Impacts Osteoarthritis Risk. *Cell* **181**, 362-381 e328, doi:10.1016/j.cell.2020.02.057 (2020).

11 Pregizer, S. & Mortlock, D. P. Control of BMP gene expression by long-range regulatory elements. *Cytokine Growth Factor Rev* **20**, 509-515, doi:10.1016/j.cytogfr.2009.10.011 (2009).

12 Gruneberg, H. & Lee, A. J. The anatomy and development of brachypodism in the mouse. *J Embryol Exp Morphol* **30**, 119-141 (1973).

13 Langer, L. O., Jr., Cervenka, J. & Camargo, M. A severe autosomal recessive acromesomelic dysplasia, the Hunter-Thompson type, and comparison with the Grebe type. *Hum Genet* **81**, 323-328, doi:10.1007/BF00283684 (1989).

14 Storm, E. E. & Kingsley, D. M. Joint patterning defects caused by single and double mutations in members of the bone morphogenetic protein (BMP) family. *Development* **122**, 3969-3979 (1996).

15 Storm, E. E. *et al.* Limb alterations in brachypodism mice due to mutations in a new member of the TGF beta-superfamily. *Nature* **368**, 639-643, doi:10.1038/368639a0 (1994).

16 Thomas, J. T. *et al.* A human chondrodysplasia due to a mutation in a TGF-beta superfamily member. *Nat Genet* **12**, 315-317, doi:10.1038/ng0396-315 (1996).
17 Polinkovsky, A. *et al.* Mutations in CDMP1 cause autosomal dominant brachydactyly type C. *Nat Genet* **17**, 18-19, doi:10.1038/ng0997-18 (1997).
18 Holder-Espinasse, M. *et al.* Angel shaped phalangeal dysplasia, hip dysplasia, and positional teeth abnormalities are part of the brachydactyly C spectrum associated with CDMP-1 mutations. *J Med Genet* **41**, e78, doi:10.1136/jmg.2003.013904 (2004).
19 Schwabe, G. C. *et al.* Brachydactyly type C caused by a homozygous missense mutation in the prodomain of CDMP1. *Am J Med Genet A* **124A**, 356-363, doi:10.1002/ajmg.a.20349 (2004).
20 Seemann, P. *et al.* Activating and deactivating mutations in the receptor interaction site of GDF5 cause symphalangism or brachydactyly type A2. *J Clin Invest* **115**, 2373-2381, doi:10.1172/JCI25118 (2005).
21 Li, H. A statistical framework for SNP calling, mutation discovery, association mapping and population genetical parameter estimation from sequencing data. *Bioinformatics (Oxford, England)* **27**, 2987-2993, doi:10.1093/bioinformatics/btr509 (2011).
22 Hatzikotoulas, K. *et al.* Genome-wide association study of developmental dysplasia of the hip identifies an association with GDF5. *Commun Biol* **1**, 56, doi:10.1038/s42003-018-0052-4 (2018).
23 Sun, Y. *et al.* A common variant of ubiquinol-cytochrome c reductase complex is associated with DDH. *PLoS One* **10**, e0120212, doi:10.1371/journal.pone.0120212 (2015).
24 Liu, Y. *et al.* Chromatin accessibility landscape of articular knee cartilage reveals aberrant enhancer regulation in osteoarthritis. *Sci Rep* **8**, 15499, doi:10.1038/s41598-018-33779-z (2018).
25 Chan, Y. F. *et al.* Adaptive evolution of pelvic reduction in sticklebacks by recurrent deletion of a Pitx1 enhancer. *Science* **327**, 302-305, doi:10.1126/science.1182213 (2010).
26 Infante, C. R., Park, S., Mihala, A. G., Kingsley, D. M. & Menke, D. B. Pitx1 broadly associates with limb enhancers and is enriched on hindlimb cis-regulatory elements. *Dev Biol* **374**, 234-244, doi:10.1016/j.ydbio.2012.11.017 (2013).
27 Nemec, S. *et al.* Pitx1 directly modulates the core limb development program to implement hindlimb identity. *Development* **144**, 3325-3335, doi:10.1242/dev.154864 (2017).
28 Szeto, D. P. *et al.* Role of the Bicoid-related homeodomain factor Pitx1 in specifying hindlimb morphogenesis and pituitary development. *Genes Dev* **13**, 484-494, doi:10.1101/gad.13.4.484 (1999).
29 Wang, J. S., Infante, C. R., Park, S. & Menke, D. B. PITX1 promotes chondrogenesis and myogenesis in mouse hindlimbs through conserved regulatory targets. *Dev Biol* **434**, 186-195, doi:10.1016/j.ydbio.2017.12.013 (2018).
30 Sanna, S. *et al.* Common variants in the GDF5-UQCC region are associated with variation in human height. *Nat Genet* **40**, 198-203, doi:10.1038/ng.74 (2008).
31 Styrkarsdottir, U. *et al.* GWAS of bone size yields twelve loci that also affect height, BMD, osteoarthritis or fractures. *Nat Commun* **10**, 2054, doi:10.1038/s41467-019-09860-0 (2019).
32 Shungin, D. *et al.* New genetic loci link adipose and insulin biology to body fat distribution. *Nature* **518**, 187-196, doi:10.1038/nature14132 (2015).
33 Pickrell, J. K. *et al.* Detection and interpretation of shared genetic influences on 42 human traits. *Nat Genet* **48**, 709-717, doi:10.1038/ng.3570 (2016).

34 Meng, W. *et al.* Genome-wide association study of knee pain identifies associations with GDF5 and COL27A1 in UK Biobank. *Commun Biol* **2**, 321, doi:10.1038/s42003-019-0568-2 (2019).

35 Tachmazidou, I. *et al.* Identification of new therapeutic targets for osteoarthritis through genome-wide analyses of UK Biobank data. *Nat Genet* **51**, 230-236, doi:10.1038/s41588-018-0327-1 (2019).

36 Miyamoto, Y. *et al.* A functional polymorphism in the 5' UTR of GDF5 is associated with susceptibility to osteoarthritis. *Nat Genet* **39**, 529-533, doi:10.1038/2005 (2007).

37 Lotta, L. A. *et al.* Association of Genetic Variants Related to Gluteofemoral vs Abdominal Fat Distribution With Type 2 Diabetes, Coronary Disease, and Cardiovascular Risk Factors. *JAMA* **320**, 2553-2563, doi:10.1001/jama.2018.19329 (2018).

38 Vaes, R. B. *et al.* Genetic variation in the GDF5 region is associated with osteoarthritis, height, hip axis length and fracture risk: the Rotterdam study. *Ann Rheum Dis* **68**, 1754-1760, doi:10.1136/ard.2008.099655 (2009).

39 Williams, F. M. *et al.* GDF5 single-nucleotide polymorphism rs143383 is associated with lumbar disc degeneration in Northern European women. *Arthritis Rheum* **63**, 708-712, doi:10.1002/art.30169 (2011).

40 Mu, J., Ge, W., Zuo, X., Chen, Y. & Huang, C. Analysis of association between IL-1beta, CASP-9, and GDF5 variants and low-back pain in Chinese male soldier: clinical article. *J Neurosurg Spine* **19**, 243-247, doi:10.3171/2013.4.SPINE12782 (2013).

41 Posthumus, M. *et al.* Components of the transforming growth factor-beta family and the pathogenesis of human Achilles tendon pathology--a genetic association study. *Rheumatology (Oxford)* **49**, 2090-2097, doi:10.1093/rheumatology/keq072 (2010).

42 Ge, W., Mu, J. & Huang, C. The GDF5 SNP is associated with meniscus injury and function recovery in male Chinese soldiers. *Int J Sports Med* **35**, 625-628, doi:10.1055/s-0033-1355417 (2014).

43 Xiao, J. L., Meng, J. H., Gan, Y. H., Zhou, C. Y. & Ma, X. C. Association of GDF5, SMAD3 and RUNX2 polymorphisms with temporomandibular joint osteoarthritis in female Han Chinese. *J Oral Rehabil* **42**, 529-536, doi:10.1111/joor.12286 (2015).

44 Zhang, L. *et al.* A new method for estimating effect size distribution and heritability from genome-wide association summary results. *Hum Genet* **135**, 171-184, doi:10.1007/s00439-015-1621-y (2016).

45 Chen, B., Li, B., Qi, Y. J., Tie, K. & Chen, L. B. Association study between growth differentiation factor 5 polymorphism and non-contact anterior cruciate ligament rupture in Chinese Han population. *Int J Clin Exp Med* **8**, 22484-22490 (2015).
