# Supplementary material for: Joint disease-specificity at the regulatory base-pair level
Source: Nat Commun. 2021 Jul 6;12:4161. doi: 10.1038/s41467-021-24345-9 (PMC8260791; doi:10.1038/s41467-021-24345-9)
Supplement: Supplementary file 3 — Description of Additional Supplementary Files [file 41467_2021_24345_MOESM3_ESM.pdf]

## Description of Additional Supplementary Files

File Name: Supplementary Data 1
Description: GWAS Summary Table on Musculoskeletal Diseases and Traits; Modularity Analyses. Sheet 1 (GWAS CATALOG-CENTRAL CONSOL)- Columns A-B: MESH ID and corresponding label for a given GWAS entry. C: GWAS database providing a given GWAS entry. D-F: Database accession and annotated study name for given trait/disease. G-I: hg38 coordinates for a single GWAS variant. K: Reported p-value for a given GWAS variant, when reported in the database. Sheet 2 (PER LOCUS DISEASE ASSOCIATION). Description of gene windows intersected with aggregated GWAS data presented in Sheet 1. Column A: Gene TSS around which a 200kb window (100kb up/downstream) was defined. B: Number of GWAS variants (pooled across different GWAS datasets) falling within this window. C: Number of unique GWAS variants falling within this window. D: Number of unique MESH IDs associated with GWAS variants falling within this window. E: MESH IDs. For recurrent disease-risk loci, multiple MESH IDs are collapsed with '@' separator. F: MESH ID labels. Sheet 3 (PER LOCUS ATAC MODULARITY): defining gene windows for which ATAC-seq peaks were intersected. A: Gene TSS around which a 200kb window (100kb up/downstream) was defined. B: Number of merged ATAC-seq peaks falling within this window. C: Number of singleton (represented in just one tissue) ATAC-seq peaks in window. D: Number of multi-tissue ATAC-seq peaks in window. E: Number of pleiotropic (represented in all tissues) ATAC-seq peaks in window. F: Classification of window as MODULAR or NOT_MODULAR (see Methods). Sheet 4 (MODULAR GENES DISEASE LOCI): Gene windows intersected by at least one GWAS variant which were defined as MODULAR (in Sheet 3). A: Gene TSS around which a 200kb window (100kb up/downstream) was defined. B: Number of merged ATAC-seq peaks falling within this window. C: Number of singleton (represented in just one tissue) ATAC-seq peaks in window. D: Number of multi-tissue ATAC-seq peaks in window. E: Number of pleiotropic (represented in all tissues) ATAC-seq peaks in window. F: Classification of window as MODULAR or NOT_MODULAR. G: Number of GWAS variants (pooled across different GWAS datasets) falling within this window. H: Number of unique GWAS variants falling within this window. I: Number of unique MESH IDs associated with GWAS variants falling within this window. J: MESH IDs. For recurrent disease-risk loci, multiple MESH IDs are collapsed with '@' separator. K: MESH ID labels. Sheet 5 (NOT_MODULAR_GENES_DISEASE_LOCI): Gene windows intersected by at least one GWAS variant which were defined as NOT_MODULAR (in Sheet 3). Columns as described for Sheet 4. Sheet 6 (MODULAR_GENES_RECURRENT_DISEASE): Gene windows for which at least two or more GWAS diseases/trait associations were intersected, which were also defined as MODULAR (in Sheet 3). Columns as described for Sheet 4. Sheet 7 (NOTMOD_GENES_RECURRENT_DISEASE): Gene windows for which at least two or more GWAS diseases/trait associations were intersected, which were also defined as NOT_MODULAR (in Sheet 3).

File Name: Supplementary Data 2
Description: Human and Mouse ATAC-seq Peak list. Sheets 1 to 5- ATAC-seq peaks from developing E67 human acetabulum, proximal femur, distal femur, proximal tibia and distal tibia. Sheets 6 to 9- ATAC-seq peaks from E15.5 mouse proximal femur, distal femur, proximal tibia and distal tibia. Column AChromosome numbers. Column B- The starting position of the peak in the chromosome. Column C-The ending position of the peak in the chromosome.

File Name: Supplementary Data 3
Description: Intersections of European and Asian DDH and knee OA GWAS with human and mouse embryonic cartilage and human knee OA patient ATAC-seq datasets. Please see "ATAC-seq peak and risk variant intersection to whittle-down the GDF5-UQCC1 DDH and knee OA association intervals" section above for more details.

File Name: Supplementary Data 4
Description: DDH patient cohort information from northeastern China and allele analyses. Please see "DDH patient rs4911178 allele and genotype analyses" section above for more details.

File Name: Supplementary Data 5
Description: OAI knee patient data. The number of rs6060369 risk alleles for subsampled individuals from the OAI cohort separated into groups was compared (see Methods). T-test statistic, group averages and (unadjusted) pvalues shown were averaged over 1000 permutations (two-sided Student's t-test, alternative of greater copies of risk allele in higher-grade/progression group), with standard errors for these values similarly reported ('sd'); headers indicate results when using n = 50, 100 and 200 subsets. Counts of alternative and risk alleles for subsampled groups are indicated. Sheet 1: 'OAI Groups' - Comparisons of risk allele counts between individuals presenting with (KL2-3) or without (KL0-1) significant OA at study entry. 'Type' refers to whether the average, first or third quartile of subsampled data were used for t-test comparisons. Sheet 2: 'OAI Progression' - Comparisons of risk allele counts between individuals separated on the basis of presentation or progression of OA status. 'NO OA': Individuals neither enter nor exit study exhibiting significant OA. 'Present OA': Individuals enter study exhibiting significant OA. 'Progress OA': Individuals progress to significant OA prior to exiting study. Columns similar to those in Sheet 1. Headers indicate both subset size and type of summary (mean, first/third quartile) used in statistical comparisons.

File Name: Supplementary Data 6
Description: Primer and Quality Control Information.
