# Supplementary material for: Joint disease-specificity at the regulatory base-pair level
Source: Nat Commun. 2021 Jul 6;12:4161. doi: 10.1038/s41467-021-24345-9 (PMC8260791; doi:10.1038/s41467-021-24345-9)
Supplement: Supplementary file 10 — Reporting Summary [file 41467_2021_24345_MOESM10_ESM.pdf]

nature research

Corresponding author(s): Terence D. Capellini and Ata M. Kiapour

Last updated by author(s): June 10th, 2021

Reporting Summary

Nature Research wishes to improve the reproducibility of the work that we publish. This form provides structure for consistency and transparency in reporting. For further information on Nature Research policies, see our Editorial Policies and the Editorial Policy Checklist.

Statistics

For all statistical analyses, confirm that the following items are present in the figure legend, table legend, main text, or Methods section.

n/a	Confirmed	
☐	☒	The exact sample size (*n*) for each experimental group/condition, given as a discrete number and unit of measurement
☐	☒	A statement on whether measurements were taken from distinct samples or whether the same sample was measured repeatedly
☐	☒	The statistical test(s) used AND whether they are one- or two-sided<br>*Only common tests should be described solely by name; describe more complex techniques in the Methods section.*
☐	☒	A description of all covariates tested
☐	☒	A description of any assumptions or corrections, such as tests of normality and adjustment for multiple comparisons
☐	☒	A full description of the statistical parameters including central tendency (e.g. means) or other basic estimates (e.g. regression coefficient) AND variation (e.g. standard deviation) or associated estimates of uncertainty (e.g. confidence intervals)
☐	☒	For null hypothesis testing, the test statistic (e.g. *F*, *t*, *r*) with confidence intervals, effect sizes, degrees of freedom and *P* value noted<br>*Give P values as exact values whenever suitable.*
☒	☐	For Bayesian analysis, information on the choice of priors and Markov chain Monte Carlo settings
☒	☐	For hierarchical and complex designs, identification of the appropriate level for tests and full reporting of outcomes
☒	☐	Estimates of effect sizes (e.g. Cohen's *d*, Pearson's *r*), indicating how they were calculated

Our web collection on statistics for biologists contains articles on many of the points above.

Software and code

Policy information about availability of computer code

Data collection	Digital Imaging and Communications in Medicine (DICOM) images were exported for measurements of following key anatomical features in Osirix MD v7.5 (Pixemo SARL, Bernex, Switzerland).<br><br>Micro CT images were also used to generate 3D models of each bone using bone segmentation process in a commercially available image processing software (Mimics v17.0, Materialise). The 3D models were then imported to 3-matic software package (v9.0, Materialise) and then co-registered together using a global n-point registration technique.<br><br>All code is provided in the materials/methods section and supplementary information document or published in Richard et al., 2020, Cell.
Data analysis	Analysis was conducted in SPSS (v27, IBM Corp., Armonk, NY).<br>Bowtie2 v2.3.2<br>MACS2 (version 2.1.1.2)<br>IDR Software (version 2.0.3)<br>bedtools version 2.29.2<br>R version 4.0.2.<br>PLINK version 1.9<br>CrossMap version 0.3.3<br>Osirix MD v7.5<br>Mimics v17.0<br>CRISPResso v1.0<br>3-matic software package v9.0

All other data analysis pipelines are provided in the material/methods and supplementary information document or published in Richard et al., 2020, Cell.

For manuscripts utilizing custom algorithms or software that are central to the research but not yet described in published literature, software must be made available to editors and reviewers. We strongly encourage code deposition in a community repository (e.g. GitHub). See the Nature Research guidelines for submitting code & software for further information.

Data

Policy information about availability of data

All manuscripts must include a data availability statement. This statement should provide the following information, where applicable:

- Accession codes, unique identifiers, or web links for publicly available datasets
- A list of figures that have associated raw data
- A description of any restrictions on data availability

Data Availability:
All the raw data used in this paper are included in the figures (2-5), Supplementary figures (1-9), supplementary Data (1-6), as well as supplementary tables (1-15). All ATAC-seq sequencing data (raw sequencing FASTQ files and processed peak bed files) have been deposited on NCBI GEO under accession code GSE153260 (https://www.ncbi.nlm.nih.gov/geo/query/acc.cgi?acc=GSE153260).

Field-specific reporting

Please select the one below that is the best fit for your research. If you are not sure, read the appropriate sections before making your selection.

[x] Life sciences [ ] Behavioural & social sciences [ ] Ecological, evolutionary & environmental sciences

For a reference copy of the document with all sections, see nature.com/documents/nr-reporting-summary-flat.pdf

Life sciences study design

All studies must disclose on these points even when the disclosure is negative.

Sample size

The sample size are decided based on prior experiments and available datasets.

For human ATAC-seq data, we utilized an N=3 per tissue/timepoint, as previously determined using ENCODE guidelines and as published in Richard et al., 2020 Cell. Sample sizes for human patient data (DDH and knee OA) were determined by the availability of human DDH patients, and through the Osteoarthritis Initiative dataset. We used a power analysis to determine the number of mice needed to example the effects of enhancer deletions or replacements. Sample sizes for all functional studies in this submission conform to those reported in Richard et al., 2020, Cell.

Data exclusions

No data points were excluded from the analyses in this submission

Replication

All experiments have been conducted with proper replications as listed in each of the Figure Legends, along within the main manuscript and methods.

For the sub-sampling allele frequency analysis comparing groups of OAI patients, randomized subsampling of N=200 individuals was performed 200 times (with replacement) for group comparisons. Replication of these test results was confirmed when using two alternative sample sizes (N = 50 and N = 100), along with calculating the first- and third-quartile values for subsampled sets.

All mouse experiments required a minimum number of independent biological replicates =5, with numbers specificed per experiment in Supplementary Table 2, here presented again:
Line, Timepoint, Wildtype, Heterozygous, Homozygous
GROW1+/- P30 7 12 12
GROW1rs4911178-A/rs4911178-+ P56 12 44 14
R4+/- P30 5 5 5
R4+/- P365 6 15 14
R4rs6060369-T/rs6060369-+ P56 6 8 6
R4rs6060369-T/rs6060369-+
(Bony Morphometry) P365 12 19 22
R4rs6060369-T/rs6060369-+ (OARSI Scoring) P365 5 10

Randomization	Randomized sub-sampling have been done for OAI imaging data to make sure the observations are valid. To assess measurement reliability, a randomly selected subset of images (20 in mouse models and 20 in human cohorts) were reanalyzed by the same examiner and two additional examiners.<br><br>For the sub-sampling allele frequency analysis, patients from the OAI dataset were separated into the following groups for comparison testing: those who presented with no/moderate-OA (KL = 0/1) (n = 1207) in either knee at baseline and all subsequent follow-ups, those with significant OA (KL >=2) in either knee at baseline (n = 1119), and those who presented with no/moderate-OA, but progressed to significant OA in either knee at the time of last follow-up in the study (n = 208)
Blinding	Morphometric measurements and assessment of osteoarthritis in mice and human samples were done blinded to the genotypes. For DDH study, assessment of measurement data was carried out without knowledge of individual sample genotype.

Reporting for specific materials, systems and methods

We require information from authors about some types of materials, experimental systems and methods used in many studies. Here, indicate whether each material, system or method listed is relevant to your study. If you are not sure if a list item applies to your research, read the appropriate section before selecting a response.

Materials & experimental systems

n/a	Involved in the study	
☐	☒	Antibodies
☐	☒	Eukaryotic cell lines
☒	☐	Palaeontology and archaeology
☐	☒	Animals and other organisms
☐	☒	Human research participants
☐	☒	Clinical data
☒	☐	Dual use research of concern

Methods

n/a	Involved in the study	
☒	☐	ChIP-seq
☒	☐	Flow cytometry
☒	☐	MRI-based neuroimaging

Antibodies

Antibodies used	PITX1 antibody for ChIP is provided in the supplementary data file 6 and also the detailed protocol was published in Richard et al., 2020, Cell. The antibody is a commonly used antibody to detect PITX1 protein (PITX1 (G-4) X antibody; Santa Cruz Biotechnology, NJ,USA; Cat# sc-271435, RRID:AB_10658969).
Validation	The antibody is a commonly used antibody to detect PITX1 protein (PITX1 (G-4) X antibody; Santa Cruz Biotechnology, NJ, USA; sc-271435). Anti-Pitx1 Antibody (G-4) is recommended for detection of Pitx1 protein of mouse, rat and human origin by Western Blot, IP, IF and ELISA; also reactive with additional species, including and canine, bovine and porcine. In our study, we have used TransCruz reagent of PITX1 (G-4) X antibody for ChIP application (sc-271435 X). Please see below website for validation information and relevant citations for the PITX1 (G-4) X: https://www.scbt.com/p/pitx1-antibody-g-4

Eukaryotic cell lines

Policy information about cell lines

Cell line source(s)	We used the Human T/C-28a2 chondrocytes developed and acquired by Mary Goldring, identifier SCC042 (Kokenyesi et al., 2000; PMID: 11097179). TC28a2 Human Chondrocyte Cell Line; Millipore Sigma, Cat#SCC042
Authentication	After receipt of this cell line from source institution, cells were passaged and used in experimental assays without additional STR authentication or mycoplasma testing.
Mycoplasma contamination	After receipt of this cell line from source institution, cells were passaged and used in experimental assays without additional STR authentication.
Commonly misidentified lines (See ICLAC register)	This is not a commonly misidentified cell line

Animals and other organisms

Policy information about studies involving animals; ARRIVE guidelines recommended for reporting animal research

Laboratory animals	This study used the following mouse lines; R4 enhancer lacZ line also called PHC21 (Harvard Genome Modification Facility); R4+/- enhancer null mouse line also called R4(R37) (Harvard Genome Modification Facility); R4rs6060369-T/+ single allelic replacement mouse line also called MC140 (Applied StemCell); The GROW1 enhancer LacZ line (Stanford Transgenic Facility); GROW1+/- enhancer null mouse line (Harvard Genome Modification Facility); GROW1rs4911178-A/+ single allelic replacement mouse line also called MC138 (Applied StemCell); C57BL/6J, wildtype (Jackson Laboratories 000664); 129X1SVJ, wildtype (Jackson Laboratories 000691);

FVB/NJ, wildtype (Jackson Laboratories 001800). All mice were housed in standard cage settings with normal 12 hour day/12 hour night light cycles and standard ambient environmental settings of 68F (20C) to 79F (26C) temperatures and 30%-70% humidity. The sex and age of all mice used in this study are listed in Supplementary Table 2 but stated here as well:
mouse line: GROW1+/-; age: P30; sex: M
mouse line: GROW1rs4911178-A/rs4911178-+; age: P56; sex: M
mouse line: R4+/-; age: P30; sex: M
mouse line: R4+/-; age: P365; sex: M
mouse line: R4rs6060369-T/rs6060369-+; age: P56; sex: M
mouse line: R4rs6060369-T/rs6060369-+; age: P365; sex: M
mouse line (histology): R4rs6060369-T/rs6060369-+; age: P365; sex: M

Wild animals

No wild animals were used in this study

Field-collected samples

No field collected samples were used in the study

Ethics oversight

All breeding, husbandry, euthanasia, and experimental protocols strictly followed IACUC-approved protocols (Capellini: 13-04-161-2) at Harvard University.

Note that full information on the approval of the study protocol must also be provided in the manuscript.

Human research participants

Policy information about studies involving human research participants

Population characteristics

For DDH patient sample collection, processing and measurements, a total of 120 femur heads from patients with DDH were collected, albeit only 113 were further studied given genotyping issues with 7 samples. All femoral heads from patients undergoing total hip arthroplasty due to DDH were obtained with signed consent. The covariate-relevant population characteristics of the human research participants have been provided in Supplementary Data 4.xlsx document. The population study characteristics are as follows: Age: 61.21 +/- 8.31 years; Sex: Female: 93/113 subjects, Male: 20/113 subjects; A allele frequency: 84.5%, G allele frequency: 15.5%; AA genotype frequency: 73.5%, AG genotype frequency: 22.1%, GG genotype frequency: 4.4%.

For ATAC-seq experiments, the human products of conception at gestational day (E) 67 were collected from late first-trimester termination through the Laboratory of Developmental Biology at the University of Washington in full compliance with the ethical guidelines of the National Institutes of Health and with the approval of the University of Washington Institutional Review Boards for the collection and distribution of human tissues for research, and Harvard University for the receipt and use of such materials. The Laboratory of Developmental Biology obtained written consent from all tissue donors. The University of Washington Birth Defects Research Laboratory was supported by NIH award number 5R24HD000836 from the Eunice Kennedy Shriver National Institute of Child Health and Human Development. Harvard University IRB determined this sample constitutes Non-Human Subjects Determination Status (Capellini: IRB16-1504). The Lead Contact (Capellini) received no federal funds (e.g., NIH) to acquire, receive, process, or utilize this sample). The human sample was briefly washed in HBSS and transported at 4°C during shipment. Upon arrival the sample was dissected under a light dissection microscope in identical fashion to all mouse samples reported above, and subjected to the ATAC-seq protocol described below, and following approved Harvard University IRB (Capellini: IRB16-1504) and COMS (Capellini: 18-103) protocols.

Recruitment

For studying DHH, first the study was approved by the human ethics committee and Institutional Review Board of the Affiliated Zhongshan Hospital of Dalian University, China. The diagnosis of hip dysplasia was made by conventional radiographs (X-Ray) and/or computed tomography (CT) with a center-edge angle of Wiberg of less than 20° measured on a well-centered antero-posterior radiograph of the pelvis. A total of 120 femur heads from patients with DDH were collected, albeit only 113 were further studied given genotyping issues with 7 samples. All femoral heads from patients undergoing total hip arthroplasty due to DDH were obtained with signed consent. The inclusion criteria as strictly executed based upon the conventional diagnostic standard of DDH. There were no self-selection bias or other biases presented since all participants were selected by random sampling method from all DHH patients with signed consent.

Ethics oversight

The study was approved by the human ethics committee and Institutional Review Board of the Affiliated Zhongshan Hospital of Dalian University, China.

The human products of conception at gestational day (E) 67 were collected from late firsttrimester termination through the Laboratory of Developmental Biology at the University of Washington in full compliance with the ethical guidelines of the National Institutes of Health and with the approval of the University of Washington Institutional Review Boards for the collection and distribution of human tissues for research, and Harvard University for the receipt and use of such materials. The Laboratory of Developmental Biology obtained written consent from all tissue donors. The University of Washington Birth Defects Research Laboratory was supported by NIH award number 5R24HD000836 from the Eunice Kennedy Shriver National Institute of Child Health and Human Development. Harvard University IRB determined this sample constitutes Non-Human Subjects Determination Status (Capellini: IRB16-1504). The Lead Contact (Capellini) received no federal funds (e.g., NIH) to acquire, receive, process, or utilize this sample).

Note that full information on the approval of the study protocol must also be provided in the manuscript.

Clinical data

Policy information about clinical studies

All manuscripts should comply with the ICMJEguidelines for publication of clinical research and a completedCONSORT checklist must be included with all submissions.

Clinical trial registration	NCT00080171 (Osteoarthritis Initiative)
Study protocol	This DDH study was not a clinical trial. All protocols are described below as well as in Supplemental Information document.
Data collection	For studying human knee osteoarthritis, we used the OAI dataset consisting of 4,129 individuals, identified as either being at risk of developing, or suffering from, osteoarthritis. This set contains groups of 3,366 Caucasian and 763 Black/African American individuals (on the basis of self-reported ethnicity); in order to control for potential effects of demographic history, only Caucasian individuals were considered for genetic analyses (see below). This group ranged in age from 45-79 (average of 62), BMI from 17.6 to 46.8 (average of 28), consisting of 1,498 males and 1,868 females. Knee OAI genetic analyses: Genotyping data for study participants in the OAI dataset was obtained through the database of Genotypes and Phenotypes (dbGaP) with appropriate permissions (n = 4,129); self-identified race was used to separate the dataset into White/Caucasian (n = 3,366) and Black/African-American (n = 763) groups. Given the disparate sampling sizes of these two groups, and to avoid potential demographic signals on genotype, only individuals in the White/Caucasian group were further analyzed.<br><br>For studying DHH, first the study was approved by the human ethics committee and Institutional Review Board of the Affiliated Zhongshan Hospital of Dalian University, China. A total of 120 femur heads from patients with DDH were collected, albeit only 113 were further studied given genotyping issues with 7 samples. All femoral heads from patients undergoing total hip arthroplasty due to DDH were obtained with signed consent. The diagnosis of hip dysplasia was made by conventional radiographs (X-Ray) and/or computed tomography (CT) with a center-edge angle of Wiberg of less than 20° measured on a well-centered antero-posterior radiograph of the pelvis. Femoral head samples went through genotyping using Sanger sequencing.
Outcomes	For morphologic assessment of OAI patients, the primary outcomes included the bone morphological features of the femoral condyles and tibial plateau as the primary locations of morphologic differences in our mice models. Our secondary outcomes included various morphological features of the ACL and menisci as the important soft tissue structures of the knee which have shown to be involved in OA pathogenesis.<br><br>While not a clinical trial, the primary/secondary outcomes for the DDH study involved ascertaining patient genotype at GDF5 rs4911178 and collecting measurements taken on radiographs which were obtained from a patient database on DDH.
